# The physiological mTOR complex 1 inhibitor DDIT4 mediates therapy resistance in glioblastoma

**DOI:** 10.1038/s41416-018-0368-3

**Published:** 2019-02-12

**Authors:** Martha Foltyn, Anna-Luisa Luger, Nadja I. Lorenz, Benedikt Sauer, Michel Mittelbronn, Patrick N. Harter, Joachim P. Steinbach, Michael W. Ronellenfitsch

**Affiliations:** 1 Dr. Senckenberg Institute of Neurooncology, University Hospital Frankfurt, Goethe University, Frankfurt am Main, Germany; 2 University Cancer Center Frankfurt (UCT), University Hospital Frankfurt, Goethe University, Frankfurt am Main, Germany; 3 German Cancer Consortium (DKTK), Partner Site Frankfurt/Mainz, Frankfurt am Main, Germany; 4 Frankfurt Cancer Institute (FCI), University Hospital Frankfurt, Goethe University, Frankfurt am Main, Germany; 5 Department of Anatomic and Molecular Pathology and Luxembourg Centre of Neuropathology (LCNP), Dudelange, Luxembourg; 6 Luxembourg Centre for Systems Biomedicine (LCSB), University of Luxembourg, Luxembourg, Luxembourg; 7 NORLUX Neuro-Oncology Laboratory, Luxembourg Institute of Health (LIH), Luxembourg, Luxembourg; 8 Laboratoire national de santé (LNS), Department of Anatomic and Molecular Pathology, Dudelange, Luxembourg; 9 Institute of Neurology (Edinger-Institute), University Hospital Frankfurt, Goethe University, Frankfurt am Main, Germany

**Keywords:** CNS cancer, Mechanisms of disease, Molecular medicine, CNS cancer

## Abstract

**Background:**

Despite significant advances in the understanding of glioblastoma genetics and biology, survival is still poor. Hypoxia and nutrient depletion in the tumour microenvironment induce adaptive signalling and metabolic responses, which can influence sensitivity to therapeutic regimens. DNA damage-inducible transcript 4 (DDIT4) is a protein induced by hypoxia and in response to DNA stress. Mechanistically, DDIT4 inhibits mammalian target of rapamycin complex 1 (mTORC1) signalling by activation of the tuberous sclerosis 1/2 (TSC1/2) complex.

**Methods:**

Using short hairpin RNA-mediated gene suppression as well as doxycycline-regulated gene induction, we developed a glioblastoma cell model to study effects of DDIT4 under conditions of the glioblastoma microenvironment and therapy.

**Results:**

We found an intact DDIT4-mTORC1 signalling axis in human glioblastoma cells that was inducible by hypoxia. Temozolomide and radiotherapy also induced DDIT4 and repressed mTORC1 activity in some glioblastoma cell lines. DDIT4 gene suppression sensitised glioma cells towards hypoxia-induced cell death, while DDIT4 overexpression protected them. Additionally, in clonogenic survival analyses, DDIT4 induction conferred protection from radiotherapy and temozolomide, while DDIT4 gene suppression sensitised cells.

**Conclusions:**

We identified DDIT4 as a cell-intrinsic regulator for adaptive responses and therapy resistance in glioblastoma cells which may interfere with cell death induction by temozolomide, radiotherapy or hypoxia by inhibiting mTORC1 activity.

## BACKGROUND

With an incidence of ~3/100,000 glioblastoma (GB) is the most common primary malignant brain tumour in adults.^1^ Although it has received much attention in clinical and laboratory research, the prognosis of GB patients is still sobering with a median survival of <1 year in unselected cohorts.^2^ The current standard therapy is palliative and consists of surgical resection followed by radiotherapy and chemotherapy with temozolomide.^3^ Therapeutic options for recurrent disease are limited and frequently involve nitrosoureas.^4^ Bevacizumab, a humanised monoclonal antibody targeting vascular endothelial growth factor, has failed to prolong overall survival in pivotal phase III trials, possibly because it can lead to local hypoxia by inhibiting tumour-associated angiogenesis.^5,6^ An important reason for the dismal prognosis of GB is therapy resistance. In more than half of GBs, transcription of the DNA repair enzyme O6-methylguanine-DNA-methyltransferase (MGMT) from an unmethylated gene promoter renders tumour cells resistant to temozolomide.^7^ Acquired resistance to temozolomide can involve defects in the DNA mismatch repair pathway reducing susceptibility to apoptosis.^8^ Adaptation to conditions of the tumour micromilieu is a selective advantage mediated by cellular sensors. DNA damage-inducible transcript 4 (DDIT4) was first described in 2002 as a protein induced by hypoxia and DNA damage.^9,10^ Additional modes of regulation have been reported for other factors such as energy stress, reactive oxygen species and dexamethasone.^11^ Mammalian target of rapamycin complex 1 (mTORC1) has been identified as a major downstream target inhibited by DDIT4.^12^ Mechanistically, DDIT4 relieves inhibition of the tuberous sclerosis 1/2 (TSC1/TSC2) complex which in its active form is a negative regulator of mTORC1.^13^ mTORC1 is a multiprotein complex and a major regulator of cell growth, translation and metabolism.^14^ One important downstream target of mTORC1 is ribosomal protein S6 (RPS6), a component of the 40S ribosomal subunit. mTORC1 phosphorylates and activates RPS6 kinase 1 (S6K1), which then phosphorylates RPS6.

We have previously shown that activation of mTORC1 signalling correlates with sensitivity to hypoxia-induced cell death offering one potential explanation for the inefficacy of mTORC1 inhibitors in GB clinical trials.^15–17^ In the current study, we assessed the role of DDIT4 as a mediator of metabolic adaptation and therapy resistance. We report that DDIT4 mediates GB cell protection from nutrient and oxygen deprivation as well as from temozolomide chemotherapy and radiotherapy, and therefore is a potential candidate for therapeutic inhibition.

## Materials and methods

### Reagents, cell lines and culture conditions

All reagents not specified otherwise were obtained from Sigma (Taufkirchen, Germany). LNT-229, LN-308 cells (both MGMT-methylated) and G55 cells (MGMT-unmethylated) have been described.^16^ Cell lines were cultured in Dulbecco’s modified Eagle's medium (DMEM) containing 10% foetal calf serum (FCS) (Biochrom KG, Berlin, Germany), 100 IU/ml penicillin and 100 µg/ml streptomycin (Life Technologies, Karlsruhe, Germany). For selection, 2 µg/ml puromycin, 400 µg/ml G418 or 50 µg/ml hygromycin B (Toku-E, Bellingham, USA) were added. When comparing different sub-cell lines, equal cell densities were confirmed by crystal violet (CV) staining in a parallel assay.^15^

### Generation of DDIT4 gene-suppressed cells

The pSUPER plasmid with the puromycin resistance cassette has been described.^15^ For stable short hairpin RNA (shRNA)-mediated gene suppression, DDIT4-specific oligonucleotide sequences were cloned into the *Bgl*II and *Sal*I sites (GATCCCCGATACTCACTGTTCATGAATTCAAGAGATTCATGAACAGTGAGTATCTTTTTGGAAA (sense) and TCGATTTCCAAAAAGATACTCACTGTTCATGAATCTCTTGAATTCATGAACAGTGAGTATCGGG (antisense)). Stable sub-cell lines were established by transfecting cells with the pSuper DDIT4sh or a pSuper plasmid with a non-targeting control sequence (NTsh) using Attractene (Qiagen, Venlo, The Netherlands) transfection reagent.

### Generation of stable and inducible DDIT4-overexpressing cells

The pcDNA3 or pcDNA3 HA-DDIT4 plasmids were a gift of James Brugarolas and were used for DDIT4 overexpression.^12^ G55 cells transfected with the pTet-Off plasmid (Clontech, #631017) were a gift of Till Acker (Gießen, Germany) and have been described.^18^ The HA-DDIT4 sequence was cloned into the pTRE2 hygro plasmid for inducible expression. This plasmid was then transfected in G55 cells with the pTet-off plasmid. 1 µg/ml of doxycycline was added to the medium for suppression of DDIT4 gene transcription. For DDIT4 induction, doxycycline was omitted from the medium 24 h prior to experiments, whereas for the control condition doxycycline was continued.

### Induction of hypoxia

Experiments were performed in serum-free DMEM adjusted to 2 mM glucose under normoxia or 0.1% oxygen by incubation in Gas Pak pouches for anaerobic culture (Becton-Dickinson, Heidelberg, Germany).^16^

### RNA extraction and quantitative reverse transcription-PCR analysis

The quantitative PCR (qPCR) was carried out as described previously^16^ with the IQ5 real-time PCR system (Bio-Rad, Munich, Germany) and corresponding primer pairs (Table 1). 18S and SDHA (succinate dehydrogenase complex flavoprotein subunit A) RNA were both used for normalisation.^19^

**Table 1:** Primer pairs for qPCR analysis

Gene	Forward primer	Reverse primer
*18S*	5′-CGGCTACCACATCCAAGGAA-3′	5′-GCTGGAATTACCGCGGCT-3′
*SDHA*	5′-TGGGAACAAGAGGGCATCTG-3′	5′-CCACCACTGCATCAAATTCATG-3′
*DDIT4*	5′-GGATGGGGTGTCGTTGCCCG-3′	5′-GGCAGCTCTTGCCCTGCTCC-3′

### Immunoblot analysis

Immunoblot was performed using a standard protocol as described previously.^15,16^ Briefly, cells were harvested and protein concentration determined by Bradford assay. Proteins were separated by SDS-PAGE on 12% acrylamide gels and blotted to nitrocellulose membranes. Membranes were incubated with antibodies to DDIT4 (Proteintech #10638-1-AP), hypoxia-inducible factor-1α (HIF-1α) (BD #610959), P-S6RP (Ser240/244; Cell Signalling #2215), P-S6K1 (Thr389; Cell Signalling #9205), S6K1 (Cell Signalling #9202), P-4E-BP1 (Thr37/46, Cell Signalling #9459), 4E-BP1 (Cell Signalling #9452) or actin (Santa Cruz Biotechnology #sc-1616). The secondary anti-rabbit and anti-goat antibodies were obtained from Santa Cruz Biotechnology. Chemiluminescence was used for detection.^16^

### Cell density, viability and clonal survival analyses

For cell growth analyses 5000 cells were seeded per well of a 96-well plate. Cell density was evaluated by CV staining.^16^ Cell viability analysis by lactate dehydrogenase (LDH) release assay was performed with the Cytotoxicity Detection Kit (LDH) (Roche, Mannheim, Germany).^16^ For evaluation of clonal survival, 500 cells were seeded per well of a 6-well plate. Cells were treated with temozolomide or irradiation in DMEM with 10% FCS. After 24 h, the medium was replaced with fresh DMEM and the experiment stopped by CV staining when clones reached close proximity to neighbouring clones.^20^ Clones were counted manually under the microscope.

### Irradiation

Irradiation was administered with single doses of X-rays ranging from 2 to 10 Gy. Control cells were also brought to the irradiation chamber but not exposed to radiotherapy.^20^

### Statistical analysis

Statistical analysis was done using Microsoft Excel. To calculate *P* values a two-tailed Student's *t* test was used. Values of *p* > 0.05 were regarded not significant (NS), and values of *p* < 0.05 significant (*) and values of *p* < 0.01 highly significant (**). Values are displayed as mean ± standard deviation if not otherwise specified.

### Database analysis

Database analysis was performed using the GlioVis platform^21^ and the TCGA datasets for GB (TCGA_GBM) and low-grade gliomas (TCGA_LGG).

## Results

### DDIT4 is induced by hypoxia, temozolomide and radiation in GB cells

**Fig. 1:**
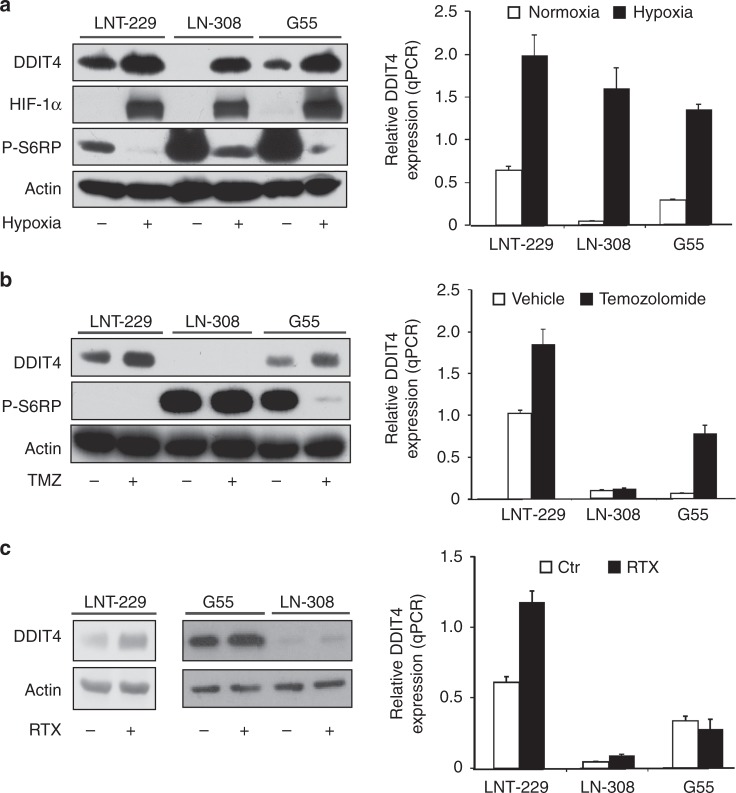
DDIT4 is induced by hypoxia, temozolomide and radiation in human glioblastoma cells. **a** LNT-229, G55 and LN-308 cells were incubated for 24 h in serum-free DMEM under normoxic conditions (21% oxygen, normoxia) or 0.1% oxygen (hypoxia). Cellular lysates were analysed by immunoblot with antibodies for DDIT4, P-S6RP, HIF-1α or actin (left panel), and cDNA was analysed for induction of DDIT4 gene expression by qPCR (right panel). **b** LNT-229, G55 and LN-308 cells were incubated for 24 h in serum-free medium containing 100 μM temozolomide (TMZ) or 100 μM DMSO (Vehicle). Cellular lysates were analysed by immunoblot with antibodies for DDIT4, P-S6RP or actin (left panel), and cDNA was analysed for induction of DDIT4 gene expression by qPCR (right panel). **c** LNT-229, G55 and LN-308 cells were irradiated (RTX+) with 10 Gy or brought to the irradiation chamber but were not placed under the X-ray beam (RTX−, Ctr). 24 h after irradiation, cellular lysates were prepared and analysed by immunoblot with antibodies for DDIT4 or actin (left panel). In a similar experiment, cells were irradiated with 10 Gy (LNT-229, LN-308) or 6 Gy (G55) and cDNA analysed for induction of DDIT4 gene expression by qPCR (right panel)

To investigate levels of DDIT4, we examined protein content in commonly used glioma cell lines as well as HEK293 cells and one colorectal carcinoma cell line (HT29) (Supplementary Fig. 1A). For our experiments, we chose LNT-229, G55 and LN-308 cells. Incubation under hypoxic conditions (0.1% oxygen), as can be found in the tumour microenvironment,^22,23^ triggered HIF-1α stabilisation and induced DDIT4 gene expression and protein with decreased phosphorylation of RPS6 as evidence for mTORC1 inhibition (Fig. 1a). Withdrawal of glucose further increased DDIT4 protein in normoxic conditions, whereas addition of 10% FCS decreased it. In hypoxic conditions, DDIT4 was induced and glucose or serum withdrawal had no additional effect (Supplementary Fig. 1B). Since DDIT4 induction has also been described in response to DNA damage in human fibroblasts,^9^ we hypothesised that temozolomide-mediated DNA damage would also induce DDIT4 in GB cells. DDIT4 induction was detectable in LNT-229 cells as well as in G55 cells exposed to temozolomide at both the RNA and protein level (Fig. 1b). Notably, this effect was dose dependent in LNT-229 cells with concomitant inhibition of mTORC1 as indicated by reduced phosphorylation of S6K1 and a dephosphorylation-mediated mobility shift in 4E-BP1 (Supplementary Fig. 1C). No regulation was detectable in p53-null LN-308 cells (Fig. 1b). Irradiation resulted in induction of DDIT4 gene expression only in LNT-229 cells; in LN-308 and G55 cells, no difference between irradiated and non-irradiated cells was detectable for DDIT4 gene expression and protein content (Fig. 1c).

### DDIT4 gene suppression sensitises human malignant glioma cells to temozolomide and irradiation

**Fig. 2:**
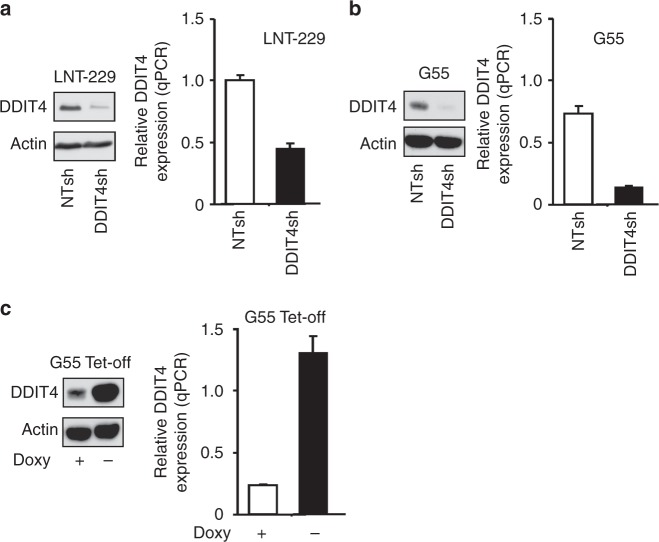
Establishment of DDIT4 gene-suppressed and gene-overexpressing glioblastoma cells. **a**, **b** LNT-229 and G55 pSuper Puro DDIT4sh and control (non-targeting sequence, NTsh) cellular lysates were analysed by immunoblot with antibodies for DDIT4 or actin. cDNA was analysed by qPCR. **c** G55 DDIT4 Tet-off cells were incubated for 3 days in serum-free DMEM with and without 1 μg/ml doxycycline. Cellular lysates were analysed by immunoblot with antibodies for DDIT4 or actin. cDNA was analysed by qPCR

**Fig. 3:**
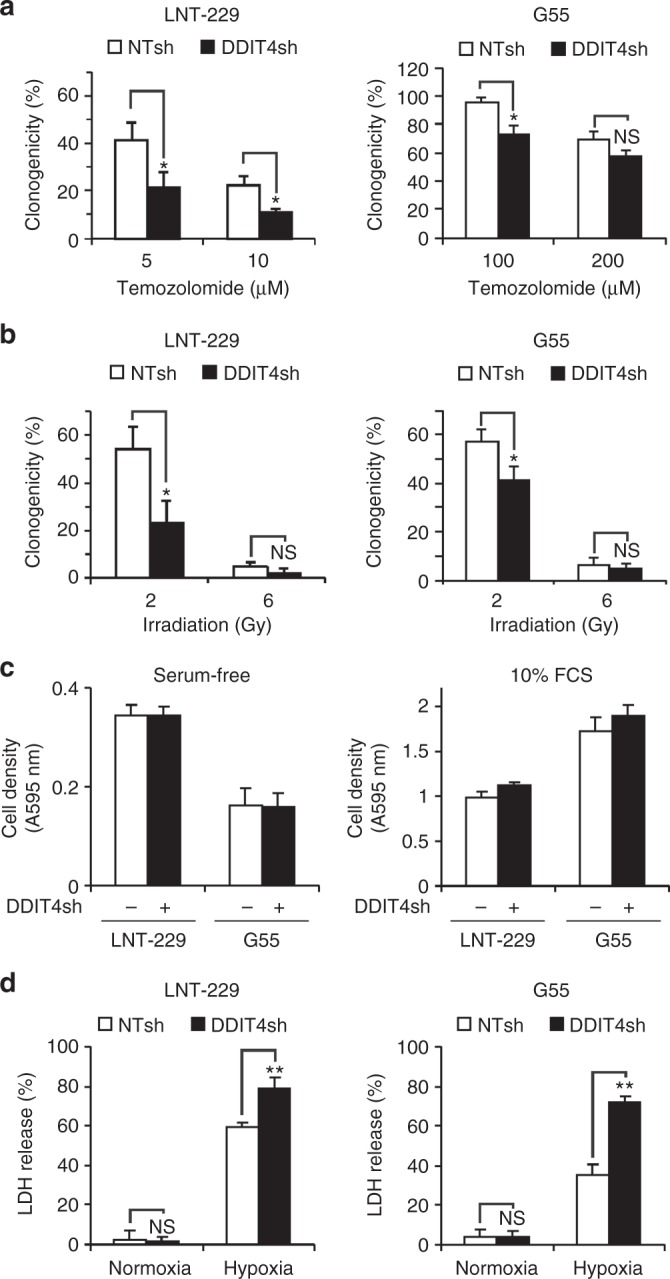
DDIT4 gene suppression sensitises human glioblastoma cells to temozolomide, irradiation and hypoxia-induced cell death. **a** LNT-229 or G55 NTsh or DDIT4sh cells were incubated for 24 h with vehicle or temozolomide as indicated. After 24 h, the medium was replaced with fresh DMEM without temozolomide. Experiments were stopped by CV staining and clones counted manually under the microscope. Clonogenicity is depicted relative to the vehicle condition (*n* = 3, mean ± S.D, NS = not significant, *p < 0.05, Student’s *t* test). **b** Cells were seeded as in **a**, irradiated with 2 or 6 Gy, and 24 h thereafter, the medium was replaced with fresh DMEM. After an incubation period of 8 days, cells were stained with CV and clones counted manually as in **a**. Clonogenicity is depicted relative to unirradiated cells (*n* = 3, mean ± SD, NS = not significant, *p < 0.05, Student’s *t* test). **c** LNT-229 or G55 NTsh and DDIT4sh cells were incubated in serum-free (left panel) or serum-containing (10% FCS, right panel) culture conditions without glucose restriction (25 mM glucose) for 4 days in normoxia. Cell density was measured by CV staining (*n* = 6). **d** LNT-229 or G55 NTsh and DDIT4sh cells were incubated for 32 h under normoxia or 0.1% oxygen (hypoxia) in serum-free DMEM with glucose restriction (2 mM glucose). Cell death was quantified by LDH release (*n* = 4, mean ± SD, NS = not significant, **p < 0.01, Student’s *t* test)

To study the effects of DDIT4 inhibition, stable sub-cell lines with DDIT4 gene suppression were generated. qPCR and immunoblot confirmed suppression of DDIT4 in LNT-229 and G55 (DDIT4sh) compared to control (NTsh) cells (Fig. 2a, b). DDIT4sh cells had increased mTORC1 signalling under basal conditions as indicated by a decreased mobility of 4E-BP1 as well as increased phosphorylation of S6K1 and RPS6 (Supplementary Fig. 1D). Notably, hypoxia- and temozolomide-mediated DDIT4 induction was also reduced in DDIT4sh cells (Supplementary Fig. 1E, F). Next, we analysed temozolomide effects on clonogenic survival and found a reduction in LNT-229 and G55 DDIT4sh cells (Fig. 3a). Further, we exposed LNT-229 and G55 cells to irradiation; again, there was an increased sensitivity with fewer surviving clones after irradiation with 2 Gy in DDIT4sh cells (Fig. 3b). Growth analyses under serum-containing (10% FCS) and serum-free culture conditions showed no clear difference in cell density in DDIT4sh cells (Fig. 3c).

### DDIT4 gene suppression sensitises human GB cells to hypoxia-induced cell death

Hypoxia is a known inducer of DDIT4 gene expression via HIF-1α-mediated transcription. This mechanism plays a key role in hypoxia-induced mTORC1 inhibition.^12^ Pharmacological or shRNA-mediated mTORC1 inhibition protects cells from hypoxia-induced cell death.^15^ We hypothesised that cells with reduced levels of DDIT4 may be less susceptible to physiological mTORC1 inhibition under hypoxia and therefore more vulnerable towards hypoxia-induced cell death as has been reported for GB cells with dysregulated mTORC1 signalling.^16^ Both LNT-229 and G55 DDIT4sh cells displayed enhanced sensitivity to hypoxia-induced cell death as indicated by an increased LDH release (Fig. 3d).

### DDIT4 confers protection against temozolomide and radiotherapy in GB cells

**Fig. 4:**
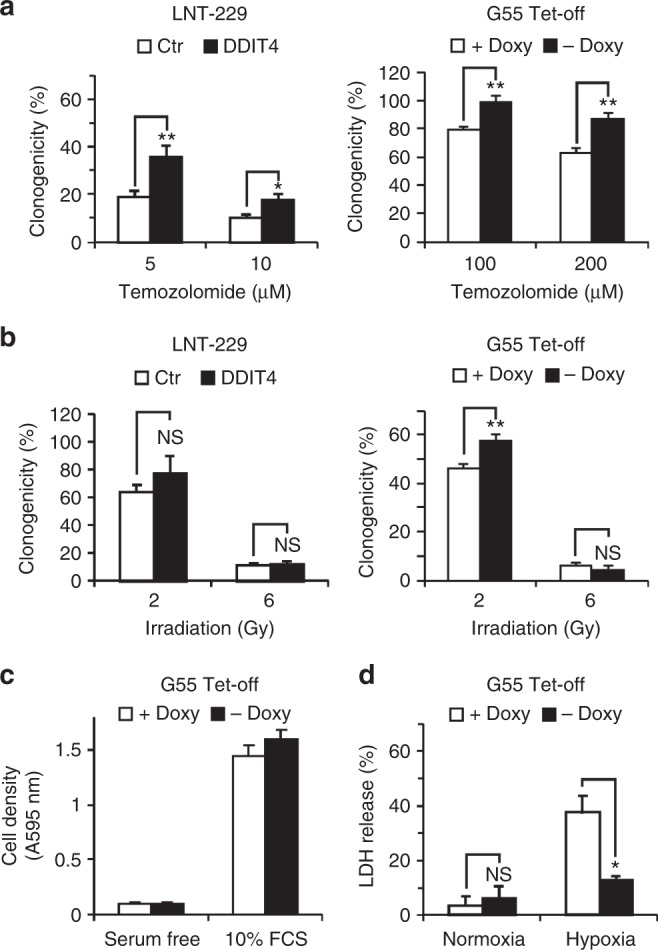
DDIT4 protects glioblastoma cells from temozolomide, irradiation and hypoxia-induced cell death. **a** LNT-229 control (Ctr, empty pcDNA3 plasmid) and HA-DDIT4-overepressing cells (pcDNA3 HA-DDIT4 plasmid) (left panel) or G55 DDIT4 Tet-off either in the presence or absence of doxycycline (right panel) were treated with temozolomide as indicated. Clonogenicity is depicted relative to the vehicle control condition (*n* = 3, mean±SD, NS = not significant, *p < 0.05, **p < 0.01, Student’s *t* test). **b** Cells were seeded as in **a** and exposed to irradiation as indicated. Clonogenicity is depicted relative to the unirradiated control condition (*n* = 3, mean ± SD, NS = not significant, **p < 0.01, Student’s *t* test). **c** G55 DDIT4 Tet-off cells were incubated in serum-free medium or DMEM with 10% FCS without glucose restriction (25 mM glucose) for 4 days under normoxia. Cell density was measured by CV staining after 4 days (*n* = 6). **d** G55 DDIT4 Tet-off cells were exposed to glucose-restricted (2 mM glucose) serum-free DMEM under normoxic conditions or 0.1% oxygen either with or without doxycycline (+Doxy and –Doxy, respectively). Cell death was quantified by LDH release (*n* = 4, mean ± SD, NS = not significant, *p < 0.05, Student’s *t* test)

Tetracycline-regulated systems allow acute induction of gene expression limiting long-term cellular adaptive or compensatory mechanisms. In G55 DDIT4 Tet-off cells, gene induction was detectable at both the mRNA and protein level when doxycycline was removed from the medium (Fig. 2c). To study the sensitivity of DDIT4-overexpressing cells to temozolomide, a clonogenicity assay was performed. Both LNT-229stable and G55 doxycycline-inducible DDIT4-overexpressing cells showed increased clonal survival, confirming a lower sensitivity to temozolomide (Fig. 4a). In conjunction with the temozolomide-mediated DDIT4 induction, these results suggest DDIT4 as a physiological resistance mechanism of tumour cells to temozolomide. Further, we exposed cells to irradiation and again G55 cells showed a lower sensitivity when DDIT4 was induced, whereas LNT-229-stable DDIT4-overexpressing cells showed only a slight trend for a survival advantage (Fig. 4b). At least for LNT-229 cells a radiation-induced DDIT4 induction had already been detected (Fig. 1c); therefore, DDIT4 is also a plausible regulator of physiological adaptation to cellular radiation damage. Notably, in LNT-229 cells, DDIT4 overexpression remained detectable over several passages (Supplementary Fig. 1G). Growth of G55 cells was not affected by DDIT4 induction (Fig. 4c).

### DDIT4 protects glioma cells from hypoxia-induced cell death

Pharmacological or shRNA-mediated mTORC1 inhibition protects cells from hypoxia-induced cell death.^15^ Furthermore, we have found that DDIT4 gene suppression sensitised GB cells to hypoxia-induced cell death (Fig. 3d). Conversely, DDIT4 overexpression protected cells from hypoxia-induced cell death (Fig. 4d).

## Discussion

While the quest for targeted therapies for GB has been unsuccessful and treatment resistance also to established drugs remains a major problem, epidermal growth factor receptor (EGFR) and mTOR remain plausible candidates as therapeutic targets. We have previously shown that pharmacological and genetic mTOR inhibition can protect human GB cells from hypoxia-induced cell death as one potential explanation for the reduced efficacy of mTOR or EGFR inhibitors in GB clinical trials in unselected patients.^15,17^ In our current study, we report a cell-intrinsic mTOR inhibitory mechanism via the regulatory protein DDIT4 that mediates mTOR-dependent therapy resistance. Notably, DDIT4 induction conferred GB cell resistance to external stimuli such as temozolomide and radiotherapy as well as tumour-intrinsic stimuli such as nutrient and oxygen deprivation (Fig. 4a, b, d). Hypoxic DDIT4 induction is therefore a potential mechanism contributing to the frequently observed reduced treatment efficacy in hypoxic tumours.^24^ Conversely, we also demonstrate that DDIT4 gene suppression sensitises GB cells to temozolomide, radiation and hypoxia-induced cell death (Fig. 3a, b, d).

Symptomatic treatment for GB frequently involves dexamethasone to reduce vasogenic oedema. It is interesting to note that dexamethasone also induced DDIT4 in LNT-229 and LN-308 cells (Supplementary Fig. 1H). This effect might contribute to the dexamethasone-mediated temozolomide resistance via the indirect mTORC2 target N-myc downstream-regulated gene 1^25^ and the evolving negative effect of dexamethasone exposure with adverse outcomes in GB patients.^26^

Potentially adverse effects of mTOR inhibition in unselected patients are highlighted by the recent results of the RTOG0913 phase II trial. This trial included 171 patients with newly diagnosed GB that were randomised to receive a daily dose of the mTORC1 inhibitor everolimus in addition to radiotherapy and temozolomide chemotherapy. Inclusion of everolimus to the treatment regimen reduced overall survival.^27^ Similar to our results with a physiological mTORC1 inhibitor, it appears possible that pharmacological mTORC1 inhibitors also render GB cells less sensitive to temozolomide (or radiation). Therefore, to effectively incorporate mTOR inhibitors in GB therapy regimes, a coordinated treatment schedule appears crucial. The results of the RTOG0913 trial argue against a (parallel) co-treatment of mTOR inhibitors with radiotherapy and temozolomide in unselected patients. However, the EORTC26082 trial that investigated the everolimus derivative temsirolimus as part of primary therapy for MGMT-unmethylated GB reported a benefit for patients with activated mTORC1 signalling in a retrospective analysis.^28^ Notably, in this trial patients were only treated with radiotherapy.

**Fig. 5:**
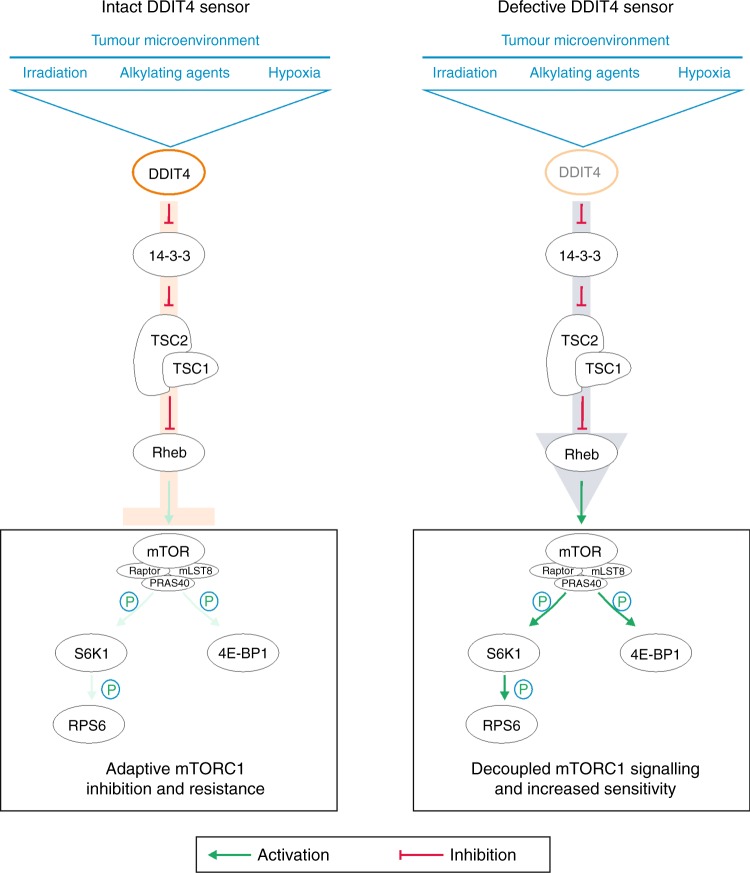
Schematic diagram illustrating DDIT4 effects on mTORC1 signalling and cellular adaptation. Irradiation, alkylating agents and hypoxia induce DDIT4 in glioblastoma cells. DDIT4 relieves 14-3-3-mediated inhibition of the TSC1/2 complex. Active TSC1/2 inhibits Rheb and ultimately mTORC1. This causes a decline in phosphorylation of the mTORC1 targets S6K1, RPS6 and 4E-BP1. An intact DDIT4 sensor effectively inhibits mTORC1 (left panel) and promotes therapy resistance. A defective DDIT4 sensor impairs induction of adaptive programmes prohibiting tumour-protective mTORC1 inhibition (right panel)

Dichotomising tumours in TCGA datasets into DDIT4 high and low groups using a median split, there was no effect on survival of GB patients (Supplementary Fig. 2A). In lower grade gliomas DDIT4 gene expression was associated with prolonged survival (Supplementary Fig. 2B). These results do not contradict our findings where DDIT4 regulates a resistance mechanism and therefore is expected to be associated with reduced patient survival. While basal DDIT4 gene expression might be beneficial in some (low-grade) tumours, it is the degree of potential adaptive upregulation of DDIT4, that is, the efficacy of an intact DDIT4 sensor to inhibit mTORC1 signalling in response to cellular stressors, that mediates therapy resistance (Fig. 5) as exemplified by the increased sensitivity of DDIT4sh cells to radiotherapy and chemotherapy (Fig. 3a, b).

In summary, we here describe DDIT4 as a mediator of cellular adaptive therapy resistance. DDIT4 conferred protection against the standard treatment regimens for GB including radiotherapy and temozolomide chemotherapy (Fig. 4a, b). Furthermore, DDIT4 also protected cells under conditions of tumour microenvironment with glucose and oxygen deprivation (Fig. 4d). Such conditions occur physiologically or can be enhanced by new treatment approaches aiming to starve tumour cells like antiangiogenic treatments. Therefore, DDIT4 is a potential target for therapeutic inhibition to disrupt tumour cell adaptation and sensitise GBs to the effects of the established treatment regimens.

## Supplementary information

Supplementary Figures 1 and 2

## Data Availability

The GlioVis platform^21^ is available online at: http://gliovis.bioinfo.cnio.es/ and the TCGA datasets for glioblastoma (TCGA_GBM) and low-grade gliomas (TCGA_LGG) (generated by the TCGA Research Network: http://cancergenome.nih.gov/) are accessible via the website.
