# Supplementary material for: The physiological mTOR complex 1 inhibitor DDIT4 mediates therapy resistance in glioblastoma
Source: Br J Cancer. 2019 Feb 12;120(5):481–7. doi: 10.1038/s41416-018-0368-3 (PMC6461855; doi:10.1038/s41416-018-0368-3)
Supplement: Supplementary file 1 — Supplementary Figures 1 and 2 [file 41416_2018_368_MOESM1_ESM.pdf]

Supplementary information

The physiological mTOR complex 1 inhibitor DDIT4 mediates therapy resistance in glioblastoma

Foltyn et al.

Supplementary figures 1 and 2 illustrate effects of DDIT4 on cell signalling and survival in different glioma cohorts.

Supplementary Fig. 1

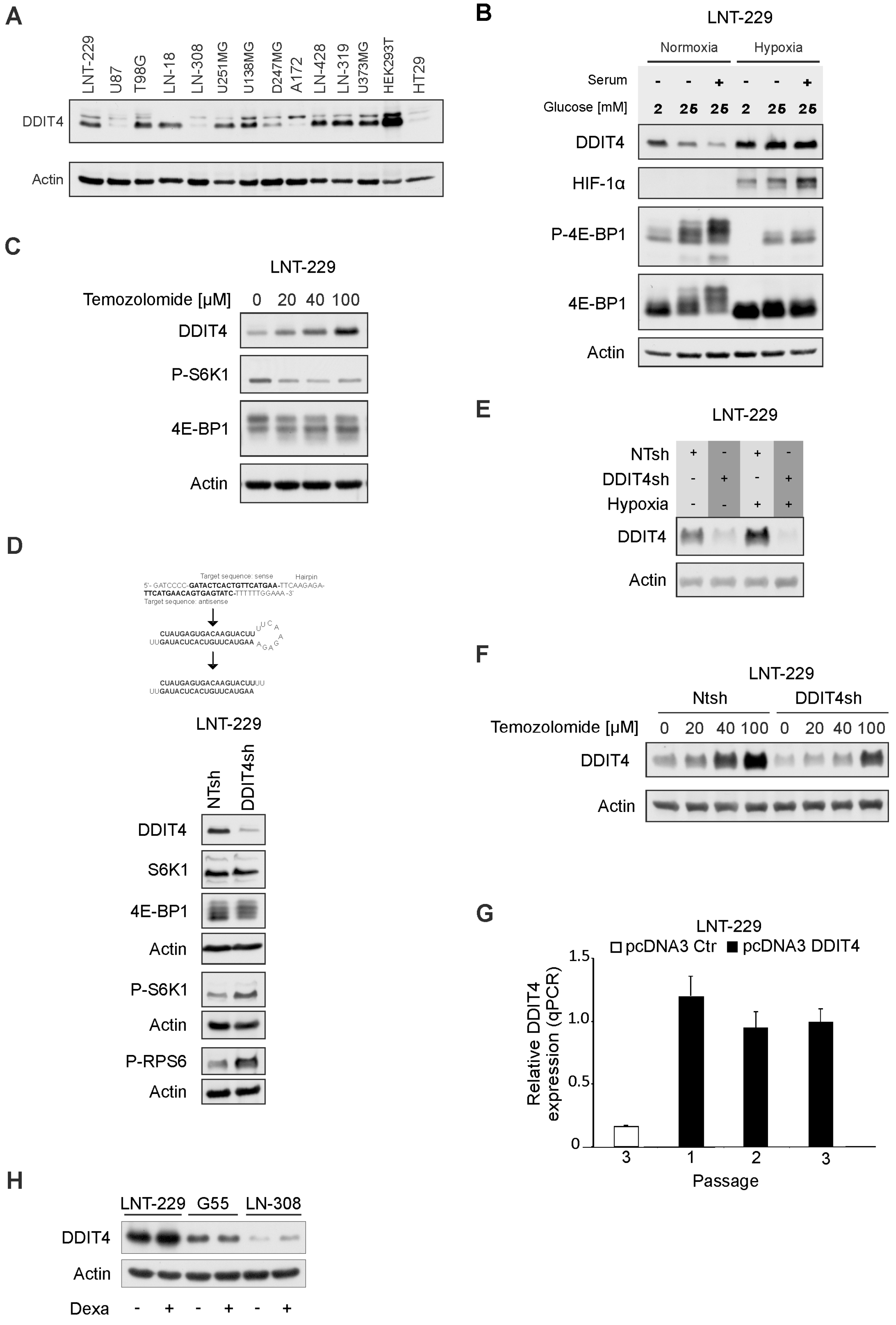

Supplementary Figure 1. DDIT4 expression and mTORC1 regulation.

A, DDIT4 protein content in common glioma cell lines (LNT-229, U87, T98G, LN-18, LN-308, U251MG, U138MG, D247MG, A172, LN-428, LN-319, U373MG), HEK293T and HT29 colon carcinoma cells. Cellular lysates were analysed by immunoblot with antibodies for DDIT4 or actin. B, LNT-229 cells were incubated in DMEM with different glucose concentrations with or without 10% FCS under normoxic or hypoxic (0.1% oxygen) conditions for 8 h as indicated. Cellular lysates were analysed with antibodies for DDIT4, HIF-1α, P-4EBP1, 4EBP1 and actin. C, LNT-229 cells were incubated in DMEM containing 10% FCS for 24 h. Cellular lysates were analysed with antibodies for DDIT4, P-S6K1, 4EBP1 and actin. D, LNT-229 NTsh and DDIT4sh cells were incubated in DMEM with 10%FCS, cellular lysates were analysed with antibodies to DDIT4, P-S6K1, S6K1, P-4EBP1, 4EBP1, P-RPS6 and actin. E, LNT-229 NTsh and DDIT4sh cells were incubated in 2 mM glucose DMEM for 8 h under normoxic or hypoxic (0.1% oxygen) conditions, cellular lysates were analysed with antibodies to DDIT4 and actin. F, LNT-229 NTsh and DDIT4sh cells were incubated in DMEM with 10%FCS for 24 h with vehicle or temozolomide as indicated, cellular lysates were analysed with antibodies to DDIT4 and actin. G, cDNA was prepared after the indicated passage number from LNT-229 cells transfected with the plasmids pcDNA3 or pcDNA3 HA-DDIT4 and analysed by QPCR. H, LNT.229, G55 and LN-308 cells were incubated for 24 h in serum free medium containing 5 µM dexamethasone (Dexa). Cellular lysates were analysed by immunoblot with antibodies for DDIT4 or actin.

Supplementary Fig. 2

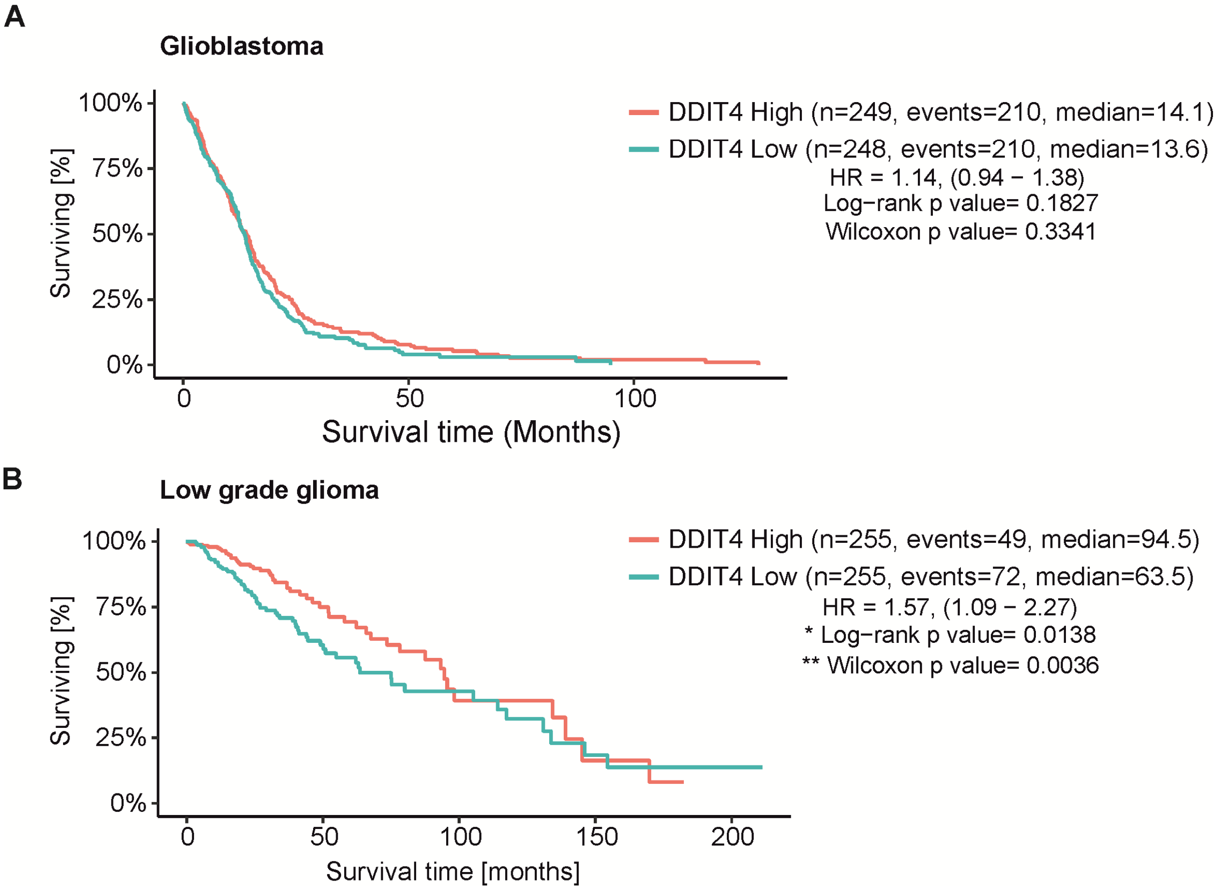

Supplementary Figure 2. Correlation analyses of DDIT4 gene expression.

A,B, correlation of DDIT4 gene expression (median split for high and low DDIT4 expressing tumours) and patient survival in the TCGA glioblastoma (primary tumours) (A) and TCGA low grade glioma (B) dataset.
